# Associations of physical activity and physical education enjoyment with self-concept domains among Hungarian adolescents

**DOI:** 10.1186/s40359-024-01953-w

**Published:** 2024-08-21

**Authors:** Tamás Berki, Tamás Csányi, László Tóth

**Affiliations:** 1 Department of Physical Education Theory and Methodology, Hungarian University of Sports Science, Budapest, 1123 Hungary; 2 Department of Psychology and Sport Psychology, Teacher Training Institute, Hungarian University of Sports Science, Budapest, 1123 Hungary

**Keywords:** Self-concept, Adolescent, Enjoyment, PACES, Physical activity, Physical education

## Abstract

**Background:**

Sport enjoyment is one of the most important factors in physical activity (PA) and physical education (PE) domains. It is not only beneficial for regular participation but also has a positive effect on mental health. Due to these benefits, this study aims to understand the relationships between PA, two forms of enjoyment, and the dimension of self-concept.

**Methods:**

The sample consisted of 315 students (M_age_=12.63). The Self-Description Questionnaire-I was used to measure the domains of self-concept. Enjoyment was measured with two scales. The Physical Activity Enjoyment Scale reflects extracurricular PA enjoyment, and the Factors Influencing Enjoyment of Physical Education Questionnaire reflects school PE enjoyment. The International Physical Activity Questionnaire was used to assess vigorous, moderate, and walking types of extracurricular PA enjoyment.

**Results:**

Hierarchical multivariate regression analysis revealed that vigorous PA predicted physical ability (β = 0.19) and physical appearance (β = 0.15). PA enjoyment was a significant predictor of general self-concept (β = 0.29), physical ability (β = 0.28), physical appearance (β = 0.16), peer relation (β = 0.16), and parental relations (β = 0.14). PE enjoyment significantly predicted general school (β = 0.17), physical ability (β = 0.27), peer relations (β = 0.21) and parental relations (β = 0.22). Furthermore, boys scored at a higher level on most of self-concept domains.

**Conclusions:**

The present study suggested that enjoyment plays a more important role in self-concept than PA. PE enjoyment mainly strengthens boys’ self-concept, but PA enjoyment is an important predictor of general self-concept in both genders. It is concluded that extracurricular PA enjoyment is beneficial, but increasing enjoyment of physical education could increase girls’ self-concepts as well.

**Supplementary Information:**

The online version contains supplementary material available at 10.1186/s40359-024-01953-w.

## Introduction

Participation in regular physical activity (PA) plays an important role in the holistic development of children and adolescents [1]. Physically active individuals not only develop stronger muscles and bones but also lay the foundation for a healthy cardiovascular system [2, 3]. In addition to physical benefits, regular exercise also plays an important role in improving cognitive function, mental health, and academic performance [4–7]. In addition to being physically active, enjoyment is a key factor for exercise participation since it can increase sport and PA participation and help adolescents engage in lifelong activities [8].

The definition of sport enjoyment was introduced by Tara Scanlan and her colleagues, who supported it as the most important factor for the sport commitment model [9–11]. According to their definition, enjoyment can be described *“as a positive affective state that reflects feelings such as pleasure*,* liking*,* and fun”* [12] (235p). Enjoyment in sports is an intrinsic motivational factor that can also be linked to another motivational model [13]. It has been well researched, and it was also adapted to the PA and physical education (PE) domains as well [14, 15].

The enjoyment of sports and PA are positively associated with various factors. Previous studies have shown that it mediates the relationship between physical literacy and participation in moderate to vigorous PA in college students [16]. PA enjoyment is also associated with the variety of PAs. For example, researchers found that high school students who perceived decreased level of PA enjoyment participated in more than one activity [17]. Furthermore, PA enjoyment is positively associated with perceived competence, academic performance, learning motivation, and social climate [18–20]. PE enjoyment is also associated with several factors as PA enjoyment does. A previous study showed that greater enjoyment of PE in early adolescence was associated with PA and self-efficacy in a school environment [21, 22]. Another study showed that the enjoyment of online PE classes during the COVID-19 lockdown was positively correlated with PA [23].

It seems that both PA and PE enjoyment relate to different mental outcomes in students, so this could also influence individuals’ self-concept. Self-concept refers to the collection of beliefs, perceptions, thoughts, feelings, and attitudes that a person has about themselves [24, 25]. Understanding the self-concept of an individual is a good way to understand and predict behavior in specific domains [26]. A widely used theoretical model of self-concept is the multidimensional and hierarchical framework of Shavelson and his colleagues [27], in which the global self-concept is at its highest level. This overarching concept can be divided into two areas: academic and nonacademic self-concept. Academic self-concept can be divided into facets related to specific school subjects, such as math, science, history, and language skills [27]. The nonacademic self-concept includes domains related to physical, social and emotional aspects, with each domain possibly further subdivided into more specific content areas. For example, physical self-concept includes subdomains such as self-perception of physical appearance and physical ability [27]. The model was further developed by Marsh [28], who recognized that the associations between the components of self-concept are weak [25, 28]. Therefore, it can be assumed that there is no single general self-concept but that several relatively independent subject- and ability-specific self-concepts exist side by side. Marsh [28] also recognized that there was little empirical support for Shavelson’s model, which is why they developed the Self-Description Questionnaire. The Self-Description Questionnaire–I serves as an instrument to assess self-concept in early adolescents [24]. It is designed to measure three dimensions of academic self-concept, namely, reading, mathematics, and general school self-concept. Four aspects of nonacademic self-concepts, namely, physical ability, physical appearance, peer relations, and parental relations, are also measured by the questionnaire. Finally, it assesses the general self-concept.

Only a few studies have investigated the relationship between enjoyment and self-concept. However, the association seems to be limited to physical domains [29–31]. For example, a study by Lohbeck and her colleagues [30] examined the specific domains of physical self-concept and found that PA enjoyment was related to endurance and global sport competence.

Gender and age differences have been previously investigated regarding sport enjoyment, physical activity and self-concept as well. Previous studies have indicated that boys tend to experience higher levels of sport enjoyment, which decrease with age [32–34]. Additionally, similar associations have been found with physical activity [33–35]. In terms of self-concept, boys appear to have higher levels of physical self-concept, including physical ability and appearance, while girls tend to have higher levels of language self-concept [36, 37]. Previous studies have emphasized the importance of adolescents as a key age group for enhancing self-concept, motivation, and physical activity [33, 38]. Therefore, this study primarily focuses on adolescents aged 10–14, as these age groups are classified as early adolescents [39, 40].

Due to the comprehensive role of self-concept, it is reasonable to believe that self-concept is related to PA and PE enjoyment. Therefore, the present study has two main objectives. Firstly, it was aimed to determine how the two types of enjoyment (PA and PE) predict the domain of self-concept. Second, it was investigated the role of PA in self-concept, as mental benefits have already been emphasized. In line with previous research, the following hypotheses are addressed: (1) Both forms of enjoyment are associated with peer relationships and physical and academic self-concept. (2) PA positively predicts the domains of physical self-concept and peer relationships (physical ability, physical appearance, peer, and parent relation). (3) Gender and age have a mediating effect on the self-concept domain.

## Methods

### Participants

Overall, 315 students ranging from 10 to 14 years of age participated in this study. The average age of the respondents was 12.63 years (SD = 1.30), and the respondents included 105 boys (M_age_ = 12.99; SD_age_ = 1.31) and 210 girls (M_age_ = 12.46; SD_age_ = 1.26). Most of the students lived in a small town (51%) with their parents (71%) in the central part of Hungary. 48% (48%) of the students participated in sports activities outside of school, and all of them had daily PE, as it is mandatory in Hungarian schools. The students were asked to rate their socioeconomic status. According to their answers, 66% of the respondents considered themselves middle-class, 27% of them upper-middle-class, and 75% of the students rated upper-class. 5% (5%) of the students reported that they were lower-middleclass. Detailed sample’s characteristic can be seen in Table 1.

**Table 1:** Description of sociodemographic characteristics of the sample

	*N*	%
Gender		
Boy	105	33%
Girl	210	67%
Family background		
Living with both parents	222	70%
Living with only with dad	11	3%
Living with only with mom	49	16%
Other	33	11%
Number of Sister/Brothers		
None	65	21%
1–2	219	69%
3 or more	31	10%
Place of Residence		
City	74	24%
Small town	160	51%
Village	81	25%
Financial status		
Upper-class	7	2%
Upper-middle-class	83	26%
Middle-class	208	66%
Lower-middle-class	17	6%
Extracurricular sports		
Yes	151	48%
No	164	52%

### Measures

#### Physical activity enjoyment scale

PA enjoyment was measured by the Hungarian version of the Physical Activity Enjoyment Scale (PACES). The scale was developed by Kendzierski and Decarlo [41] and modified by Motl and colleagues [15]. It included 16 items answered using a five-point Likert-type scale (e.g., *“When I’m active*,* I found it pleasurable”*). The answer options ranged from 1 (strongly disagree) to 5 (strongly agree). The participants were asked to think about their PAs outside of school. The questionnaire was adapted for use in Hungary in a previous study [18]. It is a unidimensional scale, and the total score can vary from 16 to 80. A higher score indicated a greater level of PA enjoyment. The scale showed excellent reliability, with a Cronbach’s alpha of 0.90.

#### Factors influencing the enjoyment of physical education

PE enjoyment was measured by the “Factors Influencing Enjoyment of Physical Education” questionnaire (PE enjoyment) [15]. The scale consisted of 12 items about possible factors that are associated with PE classes (e.g., “W*orking out with other students is something that I.“;* [15] (p. 117). It is a unidimensional scale, and the students answered on a five-point Likert-type scale (1 = Dislike a lot; 5 = Enjoy a lot). The total score could vary from 12 to 60. A higher score indicated a greater level of enjoyment in PE lessons. The scale was translated into Hungarian following the general process of scale translation [42]. A primary analysis was conducted to determine the reliability and validity of the scale since no other study investigated the construct validity of the Hungarian version. Confirmatory factor analysis showed an excellent model fit (x^2^(46) = 67.89, *p* = .20; CMIN/d.f. = 1.47; CFI = 0.98; TLI = 0.96; SRMR = 0.04; RMSEA = 0.04) and high internal reliability (Cronbach’s alpha = 0.83; average variance extracted = 0.53; construct reliability = 0.82) of the Hungarian version.

#### International physical activity questionnaire

The short form of the International Physical Activity Questionnaire (IPAQ-SF) was used in this study to assess PA. The IPAQ-SF consists of seven items, and it measures the frequency (day/week) and time (hour and minutes/day) of walking over the last seven days and moderate-(MPA) and vigorous-intensity (VPA) physical activity (e.g., “*How many days did you perform vigorous physical activity that made you feel tired or breathless during the past week*?”) [43] ( p. 2). The IPAQ-SF also measures sedentary behavior with one item, but it was not used in this study and is not part of the summary score either. The metabolic equivalent of a task (MET) was used to estimate the energy expenditure of PAs. Following the official International Physical Activity Questionnaire protocol (www.ipaq.ki.se, accessed on 21 December 2023), MET min/week values were calculated for walking and moderate- and vigorous-intensity exercise. The questionnaire was validated in Hungary earlier and has been used in a wide range of age cohorts in previous studies [44, 45].

#### Self-description questionnaire-I

Self-Concept was measured with the widely used Self-Description Questionnaire-I [46]. It consisted of 76 items, and the response categories were on a five-point Likert-type scale (1 = not at all true; 5 = very true). The scale consists of eight subscales. The subscales were as follows: Reading (10 items; self-assessment of abilities, skills, and interest in reading), Mathematics (10 items; self-assessment of abilities, skills, and interest in mathematics), General School Concept (10 items; self-assessment of abilities, skills and attitudes toward school subjects), and Physical Ability (9 items; self-assessment of abilities, skills and interest in physical activity and sports).), physical appearance (9 items; perception of the student’s own attractiveness and how their appearance appears to others), peer relation (9 items; perception of popularity among peers and how easily students make friends), parental relation (9 items; students’ perception of how they rate their relationship with their parents) and general self-concept (6 items; perception of their effectiveness and how satisfied they are with themselves). For all subscales, higher scores indicated a greater level of self-concept. The Hungarian version of the scale was translated and validated by Szenczi and Jozsa [25]. The Cronbach’s alpha values of the subscales in this study were between 0.85 and 0.92.

#### Procedure

A cross-sectional designed study was conducted in the spring of 2023. The data were collected via paper and pencil by a trained team of researchers, and the data collection took place during school lessons. Before data collection, all students were informed about the study, they were assured that their participation was voluntary, and no personal data were collected. Furthermore, all parents were informed as well, and they provided parental consent to allow their children to participate in this study. The questionnaires took approximately 10–15 min to be completed. The study was conducted in accordance with the Declaration of Helsinki, and the guidelines of the Ethics Committee at the Hungarian University of Sports Science were followed (Ethical number: MTSE-OKE-KEB/03/2023) [47].

### Statistical analysis

Statistical analysis was performed via Jamovi 2.3 for Mac. Firstly, as a primary analysis, The internal consistency and model fit indices were tested on the “Factors Influencing Enjoyment of Physical Education” scale. Then, descriptive statistics and an independent sample t-test with Cohen’s d were used to determine the sample’s characteristics and gender differences. Pearson correlations were used to measure the strengths and directions between the variables. The mean values of the psychometric questionnaires used ranged from 1 to 5 in this study. Following the primary analysis, hierarchal multiple regression was used to determine the relationships between the domains of self-concept and PA, PA enjoyment, and PE enjoyment. Each of elf-concept subscales were the outcome variable separately in this study, and the following model was tested. In the first step (Model 1), control variables, such as gender and age, were added; then, in the second step (Model 2), vigorous, moderate and walking types of PA were added. Finally, in the third step (Model 3), the variables of PACES and PE enjoyment were added.

## Results

### Descriptive statistics and correlations

Table 2 provides data on the means, standard deviations, and gender differences of the sample. VPA had the highest number (M = 1361.04; SD = 1365.72) of MET min/week. All three IPAQ-SF scores showed relatively high standard deviations. The participants reported relatively high PACES (M = 4.04; SD = 0.68) and moderate PE enjoyment (M = 3.36; SD = 0.09). Regarding the SDQ-I, general self-concept was the highest (M = 4.01; SD = 0.87), and the lowest value was for mathematics (M = 3.03; SD = 1.04). The participants showed a moderate level of most of the self-concept factors in the total sample. Gender differences were found between age, PACES, PE enjoyment, mathematics, general school concept, general self-concept, physical ability, physical appearance, and peer relation.

**Table 2:** Means, standard deviations and gender differences for the observed variables

	Total (M; SD)	Boys (M; SD)	Girls (M; SD)	t value	Cohen’s d
Age	12.63 (1.30)	12.99 (1.31)	12.46 (1.26)	3.49***	0.42
VPA (MET min/week)	1361.04 (1365.72)	1878.93 (2020.63)	1544.95 (1922.33)	1.43	0.17
MPA (MET min/week)	880.35 (942.92)	909.9 (999.27)	931.26 (1132.17)	− 0.160	− 0.02
Walking (MET min/week)	761.46 (758.98)	712.96 (786.06)	868.09 (1052.96)	-1.33	− 0.16
PACES	4.04 (0.68)	3.59 (0.71)	3.25 (0.71)	3.79***	0.48
PE Enjoyment	3.36 (0.71)	4.17 (0.65)	3.99 (0.72)	2.14*	0.27
Reading	3.66 (0.91)	3.52 (0.85)	3.7 (0.95)	-1.55	− 0.19
Mathematics	3.03 (1.04)	3.31 (0.96)	2.93 (1.09)	2.94**	0.36
General School Concept	3.15 (0.68)	3.35 (0.69)	3.04 (0.67)	3.72***	0.46
General Self-Concept	4.01 (0.73)	4.15 (0.64)	3.93 (0.76)	2.44*	0.3
Physical Ability	3.19 (0.87)	3.42 (0.76)	2.87 (0.7)	6.18***	0.75
Physical Appearance	3.38 (0.92)	3.6 (0.87)	3.27 (0.93)	2.99**	0.37
Peer Relation	3.34 (0.84)	3.61 (0.86)	3.19 (0.82)	4.00***	0.50
Parent Relation	3.93 (0.84)	3.86 (0.61)	3.77 (0.74)	1.10	0.13

Note. *p < .05; ** *p* < .01; *** *p* < .001; The mean values of PACES enjoyment, PE enjoyment and SDQ-I are standardized values

Correlation analysis between PACES scores, PE enjoyment, PA, and the domains of self-concept can be found in Table 3. PACES and PE enjoyment were positively associated with most of the variables except walking, reading, and mathematics (PA enjoyment also did not correlate with MPA). VPA was found to be positively associated with nonacademic factors related to self-concept (e.g., physical ability and peer relations). MPA was significantly associated with general self-concept, physical ability, and parental relations. The type of PA walked did not show any associations with self-concept. Age was correlated with VPA and physical appearance.

**Table 3:** Bivariate correlations for the observed variables

	1.	2.	3.	4.	5.	6.	7.	8.	9.	10	11	12	13
1. Age	—												
2. PACES	0.01	—											
3. PE Enjoyment	− 0.07	0.46***	—										
4. VPA	− 0.16**	0.34***	0.14*	—									
5. MPA	− 0.01	0.22***	0.07	0.48***	—								
6. Walking	− 0.06	− 0.00	− 0.06	0.16**	0.30***	—							
7. Reading	0.05	0.01	0.10	− 0.07	− 0.03	0.01	—						
8. Mathematics	0.03	0.10	0.11	0.05	0.08	− 0.04	0.10	—					
9. General School	0.07	0.18**	0.26***	0.03	0.04	0.01	0.47***	0.51***	—				
10. General Self-concept	0.02	0.36***	0.22	0.19***	0.13*	− 0.07	0.29***	0.19***	0.42***	—			
11. Physical Ability	0.02	0.51***	0.49***	0.38***	0.25***	0.00	0.07	0.14*	0.34***	0.41***	—		
12. Physical Appearance	0.14*	0.25***	0.17**	0.20***	0.11	− 0.08	0.05	0.10	0.24***	0.71***	0.38***	—	
13. Peer Relation	0.02	0.26***	0.33***	0.15*	0.11	0.02	0.09	0.14*	0.29***	0.57***	0.43***	0.56***	—
14. Parent Relation	0.02	0.25***	0.27***	0.15**	0.14*	− 0.08	0.20***	0.14*	0.31***	0.52***	0.27***	0.42***	0.36***

Note. *p < .05; ** *p* < .01;*** *p* < .001;

### Hierarchical multiple regression analysis

Separate analyses were conducted for each self-concept variable (Table 4). In Model 1, age and gender were added to the model as control variables in each case, and gender was found to be a significant predictor in most cases (except for reading and parent relation). PA was added to the second model. VPA was a positive predictor of general self-concept (β = 0.19), physical ability (β = 0.31), and physical appearance (β = 0.20). Walking was found to be a negative predictor of physical appearance (β=-0.13) and parental relations (β=-0.16). The final models included PACES and PE enjoyment. The PACESs were significant for general self-concept (β = 0.29), physical ability (β = 0.28), physical appearance (β = 0.16), peer relation (β = 0.16) and parental relation (β = 0.22). PE enjoyment significantly predicted general school (β = 0.17), physical ability (β = 0.27), peer relations (β = 0.21) and parental relations (β = 0.22). VPA was a significant predictor of physical appearance (β = 0.15) and physical ability (β = 0.19). The PA walking type was significantly associated with parental relationships (β=-0.14) in the final model. The variance was explained by 44% of the highest variance and 5% of the lowest variance in the models.

**Table 4:** Results of hierarchical multiple regression analysis for the predictors of self-concept

		Reading β (SE)	Mathematics β (SE)	General School β (SE)	General Self-Concept β (SE)	Physical Ability β (SE)	Physical Appearance β (SE)	Peer Relation β (SE)	Parent Relation β (SE)
**Model 1**									
	Gender^a^	0.04 (0.12)	− 0.17** (0.14)	− 0.21*** (0.09)	− 0.13*** (0.09)	− 0.33*** (0.09)	− 0.15** (0.12)	− 0.22*** (0.11)	− 0.02 (0.09)
	Age	− 0.15* (0.04)	0.03 (0.14)	0.00 (0.03)	0.04 (0.03)	− 0.08 (0.03)	0.10 (0.04)	− 0.02 (0.04)	− 0.01 (0.03)
**Model Summary**	R^2^	0.01	0.03	0.04	0.02	0.11	0.04	0.05	0.00
	ΔR^2^	0.00	0.00	0.00	0.00	0.00	0.00	0.00	0.00
	F	3.77*	4.51*	6.39**	2.96	17.02***	5.16**	7.00**	0.07
**Model 2**									
	Gender^a^	0.03 (0.12)	− 0.16** (0.14)	− 0.21*** (0.09)	− 0.10 (0.09)	− 0.30*** (0.09)	− 0.13* (0.12)	− 0.21*** (0.11)	0.00 (0.09)
	Age	− 0.17** (0.04)	0.04 (0.05)	0.01 (0.03)	0.07 (0.03)	− 0.03 (0.03)	0.13* (0.04)	0.00 (0.04)	0.01 (0.03)
	Vigorous PA	− 0.10 (0.00)	0.04 (0.00)	0.00 (0.00)	0.19** (0.00)	0.31*** (0.00)	0.20* (0.00)	0.12 (0.00)	0.10 (0.00)
	Moderate PA	− 0.02 (0.00)	0.10 (0.00)	0.04 (0.00)	0.07 (0.00)	0.12 (0.00)	0.06 (0.00)	0.08 (0.00)	0.11 (0.00)
	Walking	0.03 (0.00)	− 0.06 (0.00)	,02 (0.00)	− 0.11 (0.00)	− 0.07 (0.00)	− 0.13* (0.00)	− 0.04 (0.00)	− 0.16* (00)
**Model Summary**	R^2^	0.04	,05	0.05	0.07	0.24	0.09	0.08	0.04
	ΔR^2^	0.01	0.02	0.01	0.05	0.13	0.05	0.03	0.04
	F	2.11	2.18*	2.69*	4.44***	17.89***	5.55***	4.42***	2.57*
**Model 3**									
	Gender^a^	0.05 (0.12)	− 0.15* (0.14)	− 0.16** (0.09)	− 0.07 (09)	− 0.21*** (0.09)	− 0.10 (0.12)	− 0.15* (0.11)	0.05 (0.09)
	Age	− 0.16* (0.04)	0.04 (0.05)	0.02 (0.03)	0.08 (0.03)	− 0.01 (0.03)	0.13* (0.04)	0.02 (0.04)	0.03 (0.03)
	Vigorous PA	0.03 (0.00)	0.01 (0.00)	− 0.06 (0.00)	0.09 (0.00)	0.19*** (0.00)	0.15* (0.00)	0.03 (0.00)	0.05 (0.00)
	Moderate PA	− 0.04 (0.00)	0.10 (0.00)	0.02 (0.00)	0.04 (0.00)	0.07 (0.00)	0.04 (0.00)	0.06 (0.00)	0.10 (0.00)
	Walking	0.05 (0.00)	− 0.05 (0.00)	,05 (0.00)	− 0.09 (0.00)	− 0.03 (0.00)	− 0.11 (0.00)	0.00 (0.00)	− 0.14* (00)
	PACES	0.04 (0.09)	0.05 (0.11)	0.14 (0.07)	0.29*** (0.07)	0.28*** (0.05)	0.16* (0.09	0.16* (0.08)	0.14* (0.07)
	PE Enjoyment	0.10 (0.09)	0.05 (0.07)	0.17* (0.08)	0.05 (0.08)	0.27*** (0.05)	0.06 (0.08)	0.21** (0.08)	0.22*** (0.06)
**Model Summary**	R^2^	0.05	0.06	0.11	0.16	0.44	12	0.16	0.12
	ΔR^2^	0.01	0.01	0.06	0.09	0.20	0.03	0.08	0.08
	F	2.07*	2.16*	4.53***	7.61***	30.84***	5.27***	7.16***	5.02***

Note. *p < .05; ** *p* < .01;*** *p* < .001;^a^Gender: 1 = boys; 2 = girls

## Discussion

The purpose of this study was to understand the relationships between the domains of self-concept, PA, and two forms of enjoyment. It was hypothesized that PA would mainly predict the domains of physical self-concepts (e.g., physical ability, physical appearance), peers, and parental relationships. The forms of enjoyment were associated with physical, academic, and peer relation variables. Finally, gender and age had a mediating role in self-concept domains. The results showed that gender was predictive of most self-concept variables, but age only predicted reading and physical appearance. However, PA only predicted physical ability and physical appearance in the final model. Additionally, the findings of this study revealed that most of the self-concept variables were significantly impacted by both forms of enjoyment.

An investigation between the self-concepts of Reading and Mathematics showed that there was no single prediction regarding PA and enjoyment. Due to the positive effects of sport and PA on academic performance, it is believed that these effects might be associated with these variables [18]. The reason behind this phenomenon is that the positive effects of PA and enjoyment are not uniform and may increase overall academic self-concept but not necessarily individual self-concept [48]. Regarding the general school concept, PE enjoyment was a significant predictor, and the effects were controlled by gender, indicating that boys reported a positive increase in their attitudes, abilities, and skills related to school. This finding not only shows the importance of PE enjoyment but also suggests that there is an indirect positive effect between general school concept and PE. Hence, increasing PE enjoyment in both genders is important since it affects day-to-day mood in school settings.

Furthermore, no influence of gender on general self-concept was found if PA and enjoyment were included in the model. Nevertheless, Vigorous PA was a positive predictor of self-concept in the second model. The role of PA in self-concept was expected due to the positive mental influence of PA [49, 50], but in this study, only high levels of PA increased this effect. Another interesting finding was that if the enjoyment variables were added to the model, the role of PA decreased, and PA enjoyment became a positive predictor. These results indicate that PA enjoyment is more important than PA itself if an individual enjoys any activity, which will positively affect their efficacy and self-belief [23]. There were no gender differences in the final model that suggested that PA enjoyment had a positive effect on general self-concept. The difference between the two forms of enjoyment must be acknowledged. While PE enjoyment was a predictor of the general school concept, PA enjoyment was significantly associated with the general self-concept. It seems that PAs inside and outside of school could separate in this respect as well.

The regression result for physical ability was the strongest model. Boys with high levels of vigorous PA who enjoy both PA and PE sense higher levels of physical ability. The results were in line with previous literature and showed the importance of PA and the enjoyment of motor skills and sports [29, 51–53]. Additionally, most adolescents seem to be aware of their physical condition. In addition to physical ability, physical appearance showed another important result. Gender, age, education, and walking type were significant predictors of physical appearance in the second model. However, when enjoyment was added to the model, gender differences were equal. It was assumed that if girls enjoy sport or PA participation, the effect would be the same as that for boys. PA has an advantage in appearance since those who regularly participate in any sport or PA feel more strength and vitality [54, 55]. Age positively predicted physical appearance as well. Previous studies have shown similar results, showing that the perception of appearance increases as a part of mental development [56].

Self-concept of peer relations, which describes feelings toward peers, friends, and individual popularity, was influenced by both enjoyment and gender. The relationships between gender and peer relations are not consistent to the literature, but following Kesebir’s [57] conclusion, it is believed that girls tend to form intimate dyadic bonds, while boys interact in larger groups and compete for rank, which is an important characteristic of sports and PA [57]. The recognition of cultural differences that might influence peer relations must be included [58]. A relationship between the two types of enjoyment was also expected. Peers have a great influence on enjoyment according to Scanlan’s sport-enjoyment model [11, 59]. Consistent with relationships with peer relations, parental relations were also predicted by the two types of enjoyment. The reason for this finding is twofold. Firstly, parental support is an important factor in the sport enjoyment model [11, 59]. Secondly, experiencing enjoyment through sports or PA means less stress and frustration for the children, allowing them to build a better relationship with their parents.

Several cases of PA were negatively associated with walking type (e.g., parent relation and physical appearance). This was an unexpected finding, as several studies have shown the benefits of walking for mental health (e.g., Pascoe & Parker, 2022). The reason for this association is that children may not be aware of the positive influence of walking and think that it is just a necessary task that they have to do every day.

Overall, enjoyment seems to play a more important role in self-concept than PA. To achieve the great value of sport and PA, it is not enough to participate, and it is also important to enjoy the activity. Participation in sports also has a constrained side that has negative psychological effects (e.g., anxiety). Therefore, it is important to increase the enjoyment of PA in and out of school. Compared with boys, girls perceived lower levels of both forms of enjoyment, but increasing enjoyment could positively affect their self-concept.

Even though this study shows clear results, some limitations need to be considered. Firstly, most of the regression results showed low explained variance in the data, which might be increased by a more heterogeneous sample or by adding more schools to the study. Another limitation is that the generalizability of the findings is limited due to the use of a random sample and the fact that this study included mainly girls. Finally, the self-administered questionnaire is also a limitation since it does not provide a realistic picture of the student’s feelings. Despite these limitations, the present study yielded exciting results with potential for future replication in several areas: self-esteem, perceived competence, and goal orientation. The study demonstrates that enjoyment plays a crucial role in adolescents’ self-concept, which could in turn improve their mental health and psychological well-being.

## Conclusions

In summary, enjoyment had a stronger effect on self-concept than PA. There is also a clear difference between genders, suggesting that boys perceive a greater level of self-concept. However, this study revealed that increasing enjoyment for girls could increase their self-concept as well. PE teachers and coaches should be encouraged to make their teaching and training more fun and enjoyable, as this helps adolescents improve their skills, abilities, and attitudes in other life aspects, too. It is hoped that this study will provide useful information for researchers, teachers, and coaches working with children and adolescents.

### Electronic supplementary material

Below is the link to the electronic supplementary material.

Supplementary Material 1

## Data Availability

Upon request, the corresponding author will provide the datasets used and analyzed during the current study.

## Abbreviations

CFI: Comparative Fit Index
CMIN/d.f.: Chi square divided by the degrees of freedom
IPAQ-SF: International Physical Activity Questionnaire Short-form
M: Mean
MET: Metabolic Equivalent of a Task
MPA: Moderate Physical Activity
PA: Physical Activity
PACES: Physical Activity Enjoyment Scale
PE: Physical Education
RMSEA: Root Mean Square Error of Approximation
SD: Standard Deviations
SRMR: Standardized Root Mean Square Residual
TLI: Tucker-Lewis Index
VPA: Vigorous Physical Activity
